# Excess HB-EGF, which promotes VEGF signaling, leads to hydrocephalus

**DOI:** 10.1038/srep26794

**Published:** 2016-05-31

**Authors:** Joon W. Shim, Johanna Sandlund, Mustafa Q. Hameed, Bonnie Blazer-Yost, Feng C. Zhou, Michael Klagsbrun, Joseph R. Madsen

**Affiliations:** 1 Department of Neurosurgery, Boston Children’s Hospital and Harvard Medical School, Boston, MA 02115, USA; 2 Department of Biology, Indiana University Purdue University, Indianapolis, IN 46202, USA; 3 Vascular Biology Program, Boston Children’s Hospital and Harvard Medical School, Boston, MA 02115, USA; 4 Department of Neurology, Boston Children’s Hospital and Harvard Medical School, Boston, MA 02115, USA; 5 Department of Anatomy and Cell Biology, Indiana University School of Medicine, Indianapolis, IN 46202, USA; 6 Department of Surgery and Pathology, Boston Children’s Hospital and Harvard Medical School, Boston, MA 02115, USA; 7 Department of Medicine, Boston University School of Medicine, Boston, MA 02118, USA; 8 Department of Pathology, Stanford University School of Medicine, 300 Pasteur Drive L235, Stanford, CA 94305, USA; 9 Clinical Microbiology Laboratory, Stanford University Medical Center, 3375 Hillview Avenue Palo, Alto, CA 94304, USA

## Abstract

Heparin binding epidermal growth factor-like growth factor (HB-EGF) is an angiogenic factor mediating radial migration of the developing forebrain, while vascular endothelial growth factor (VEGF) is known to influence rostral migratory stream in rodents. Cell migratory defects have been identified in animal models of hydrocephalus; however, the relationship between HB-EGF and hydrocephalus is unclear. We show that mice overexpressing human HB-EGF with β-galactosidase reporter exhibit an elevated VEGF, localization of β-galactosidase outside the subventricular zone (SVZ), subarachnoid hemorrhage, and ventriculomegaly. In Wistar polycystic kidney rats with hydrocephalus, alteration of migratory trajectory is detected. Furthermore, VEGF infusions into the rats result in ventriculomegaly with an increase of SVZ neuroblast in rostral migratory stream, whereas VEGF ligand inhibition prevents it. Our results support the idea that excess HB-EGF leads to a significant elevation of VEGF and ventricular dilatation. These data suggest a potential pathophysiological mechanism that elevated HB-EGF can elicit VEGF induction and hydrocephalus.

Hydrocephalus, characterized by dilatation of the cerebral ventricles due to excessive accumulation of cerebrospinal fluid (CSF), is classified into congenital and acquired hydrocephalus^1^. It can develop as a complication of subarachnoid hemorrhage (SAH)^2^, which is often reported as post-hemorrhagic hydrocephalus (PHH)^3^. The PHH has several types, namely, hydrocephalus following SAH, intraventricular hemorrhage (IVH), and germinal matrix hemorrhage (GMH)^3^,^4^. Ages of patients with hydrocephalus encompass a wide spectrum: fetal to adult onset. Neural and non-neural cells including vascular endothelial cells are affected by hydrocephalus, especially, with elevated intracranial pressure^5^.

Heparin binding epidermal growth factor-like growth factor (HB-EGF) is an angiogenic growth factor of two distinct forms: membrane-bound and soluble HB-EGF. HB-EGF is localized in the ventricular zone (E13) and cortical layers (E16) during development^6^. In pathological conditions, vascular endothelial cell^7^, wound fluid^8^ and blood cell such as monocyte, macrophage, and platelet are known to release HB-EGF. In cerebral blood vessels prior to vasoconstriction, active HB-EGF is produced through phosphorylation of its receptor, epidermal growth factor receptor (EGFR) via enzymatic cleavage of the membrane-bound precursor HB-EGF^9^. The soluble HB-EGF is shown to mediate vasospastic response in parenchymal vessels. In SAH associated with intracranial aneurysm, rupture of the aneurysm evokes blood into the subarachnoid space. Coagulation of such subarachnoid blood activates platelets, which release growth factors in the wall of the vessels^10^. While HB-EGF plays a causal role in vasoconstriction of an animal model with SAH^9^, whether excess HB-EGF is involved in the pathogenesis of hydrocephalus is unknown.

HB-EGF has been implicated in migration of the forebrain cells. During neural development, cell migration allows precursors to move towards the destined location^11^,^12^. Radial migration, a mechanism that young neurons use during corticogenesis ceases after birth^12^. The rostral migratory stream (RMS), a specialized route of cell movement reported in adult rodents is tangential migration from subventricular zone (SVZ) to the olfactory bulb and continues during adulthood^13^,^14^. The SVZ, also called as subependyma is the germinal region in the adult brain with a heterogeneous cytoarchitecture contacting cerebrospinal fluid (CSF)^15^,^16^. The subependyma accounts for neurogenesis and migration of neuroblast^13^,^14^. In the prenatal forebrain, it has been suggested that developmental changes in HB-EGF regulate the cell migration by a chemoattractive mechanism^6^. Cells of ventricular explants expressing EGFR have been shown to migrate towards the soluble HB-EGF^6^. HB-EGF is also shown to induce vascular endothelial growth factor (VEGF)^18^. Consistent with this, an anti-HB-EGF monoclonal antibody, Y-142, is exhibited to inhibit VEGF protein production in the supernatant of cell culture more effectively than a VEGF inhibitor, bevacizumab^19^. Using VEGFR1 knockout mice, it has been proposed that the proper cell migration of the postnatal forebrain depends on endogenous VEGF signaling^20^. In prenatal impairments of radial migration, hydrocephalus is reported postnatally^17^ but it is yet unclear whether hydrocephalus is caused by the defective postnatal migration of neuroblasts or by other factors. While involvement of growth factors in neural migration of the forebrain is known, HB-EGF mediated hydrocephalus, particularly, in the adult brain has not been reported.

Here we hypothesize that HB-EGF affects VEGF signaling and the fluid circulation in the cerebral ventricles. In testing this hypothesis we demonstrate whether exogenous HB-EGF induces VEGF. Using mice expressing human HB-EGF, we determine the localization of HB-EGF in the postnatal brain with and without hydrocephalus. By infusing VEGF, VEGFR2 blocker, and co-infusion with VEGF ligand inhibitor in rats, we demonstrate whether VEGF receptor or ligand inhibition changes neural progenitors of the SVZ and ventriculomegaly in rats. Using a ciliopathy model with hydrocephalus independent of HB-EGF, we further test whether cell migration in either tangential or radial orientation is altered in hydrocephalus. We predict that the regulation of ventricular size and neuroblast migration in this setting and others is potentially mediated by the actions of HB-EGF to impact the signal transduction network that regulates relocation of the SVZ cells in hydrocephalus.

## Results

### HB-EGF induces endogenous VEGF

**Figure 1:**
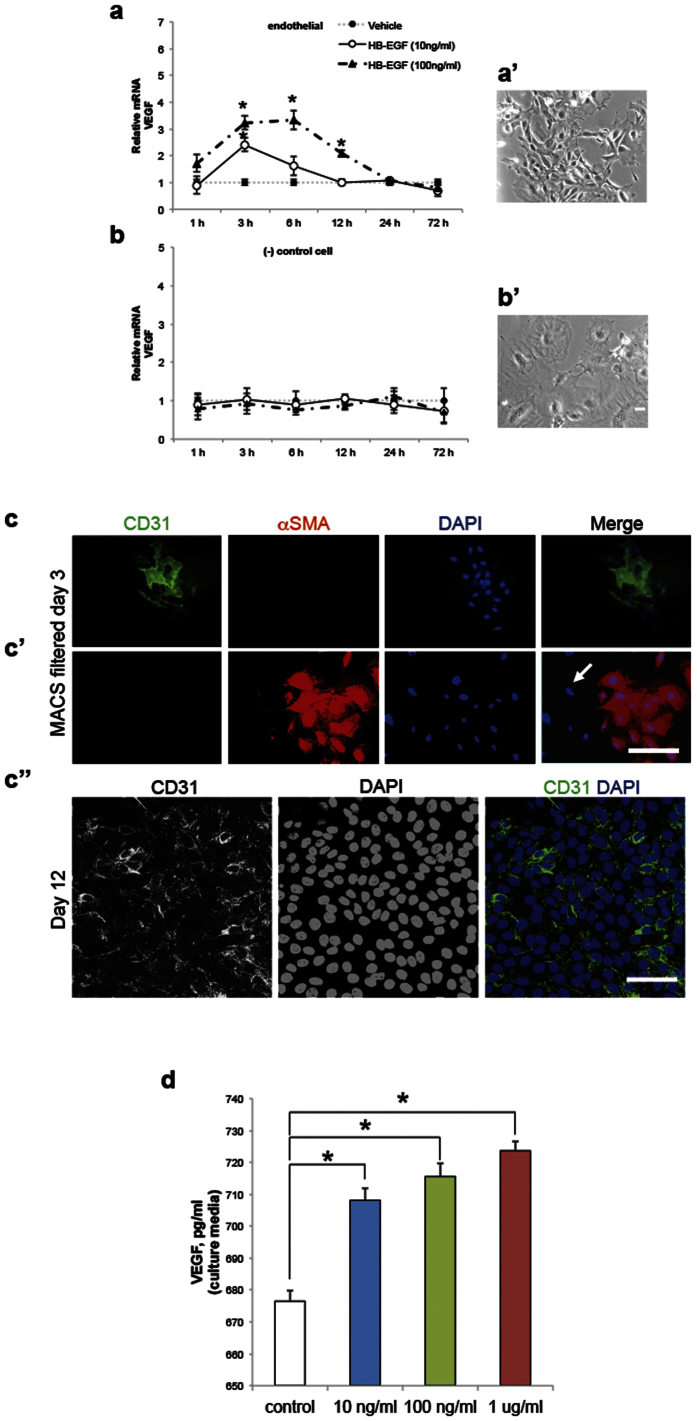
Effect of HB-EGF on endogenous VEGF *in vitro*. (**a**) VEGF mRNA expression in mouse endothelial cell (CD31+) treated with 0, 10, and 100 ng/ml of recombinant human HB-EGF. (a’) A phase-contrast micrograph displaying endothelial cell reaching approximately 70% confluent state prior to HB-EGF treatment. (**b**) VEGF mRNA expression in mouse non-endothelial cell (CD31−) treated with 0, 10, and 100 ng/ml of recombinant human HB-EGF. Error bar represents the standard error (n = 4 for 1, 3, 6, and 12 h; n = 4 for 24 and 72 h data, respectively). (b’) A phase-contrast micrograph displaying non-endothelial cell reaching approximately 70% confluent state prior to HB-EGF treatment (**c**) Micrographs displaying CD31+ mouse brain endothelial cells cultured 3 day following magnetic isolation through the manual cell separation columns (MACS). (c’) The CD31 positive cells are clustered in the absence of αSMA positive vascular smooth muscle cell. The CD31 negative cell population is heterogenous, containing αSMA positive cells along with the cellular phenotype that are negative with anti-CD31 and negative with anti-αSMA stain (arrow). (c’) A representative micrograph demonstrating CD31 positive vascular endothelial cells on day 12 of the culture following MACS based isolation used in the exogenous HB-EGF experiments. Four independent replicate experiments from four C57BL6 mice (n = 4). Scale bars, 40 μm (**c–**c’) and 20 μm (c”) (**d**) Bar graph exhibiting VEGF protein levels in culture media of mouse endothelial cell treated at 0, 10 ng, 100 ng, and 1 μg/ml of HB-EGF. Four replicate experiments (n = 4) in this figure (**d**). Single asterisk denotes a statistical significance by Dunnett’s post-hoc test with respect to the control after ANOVA (P < 0.05)

To determine whether exogenous HB-EGF leads to an endogenous VEGF expression we treated CD31+ mouse brain endothelial cells with human HB-EGF at 10 and 100 ng/ml, respectively. The mRNA expression of VEGF was significantly elevated 3 h following 10 ng/ml of HB-EGF (2.4 fold) relative to the untreated controls (p = 0.016). In a higher dose (HB-EGF = 100 ng/ml), further increases (3.2–3.5 fold) in VEGF mRNA levels were detected at three time points (3, 6 and 12 h) relative to the controls (p = 0.02, p = 0.01 and p = 0.03, respectively). In the negative controls (CD31 negative and α smooth muscle actin or αSMA positive), however, no significant change was observed (Fig. 1a,b). We then assayed the VEGF protein level in the conditioned media of CD31+ cell culture. The anti-CD31 immunoreactivity did not show a significant difference or an altered staining pattern between the vehicle control and HB-EGF (100 ng/ml) treated culture. VEGF protein was significantly elevated in the culture media of CD31+ endothelial cell treated with HB-EGF at 10 ng/ml, 100 ng/ml, and 1 μg/ml as compared to the control (vehicle treated), respectively (Fig. 1c,d). This *in vitro* experiment has shown that exogenous administration of human soluble HB-EGF induces endogenous VEGF in the brain vascular endothelial cell.

### HB-EGF infusion increases Evans blue leakage and ventricular size

**Figure 2:**
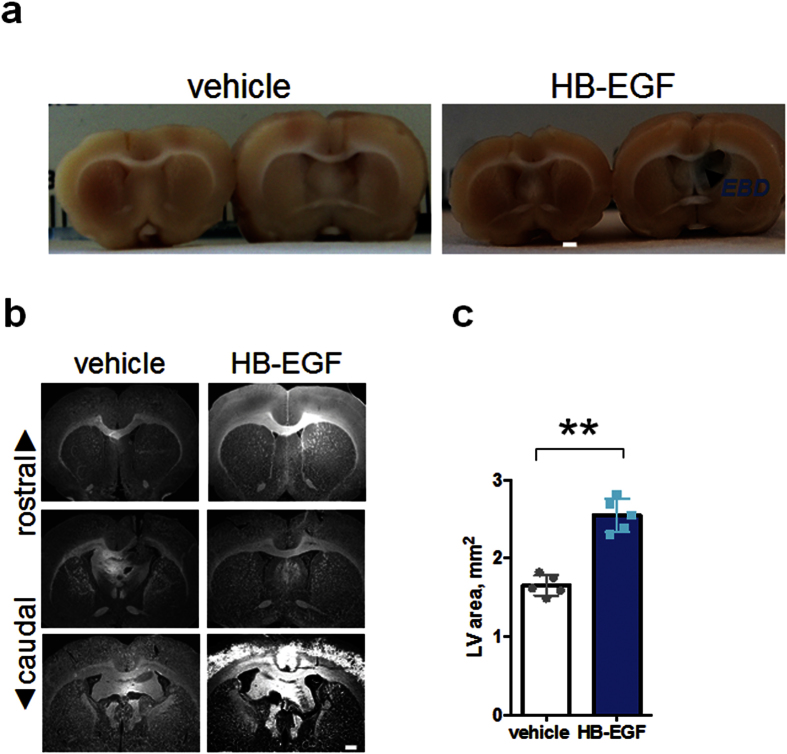
Effect of HB-EGF infusion on rat brain ventricles *in vivo*. (**a**) Photographs showing Evans blue leakage in the ipsilateral septum and subependyma of the rat brain infused with HB-EGF for 14 days at 10 μg/ml (0.5 μl/hr). Note that Evans blue as injected to the tail vein did not leak in the vehicle infused brain (left). EBD denotes Evans blue dye. Three animals per each group were used for Evans blue injection. (**b**) Coronal sections visualized with DAPI nuclear marker displaying a representative rostral and caudal brain infused with vehicle (left) and HB-EGF (right). Scale bar, 1 mm. (**c**) A bar graph with scattered data point showing a ventricular size measured by cross-sectional area of the brains infused with vehicle (left) and HB-EGF (right) for 14 days. Double asterisk, ** denotes statistical significance by unpaired t test at p = 0.01(two-tailed). n = 5 in each group for all the experiments in this figure (**b,c**).

To examine whether HB-EGF has an ability to evoke a similar effect seen in the VEGF-induced permeability, we infused human HB-EGF into the lateral ventricle of the adult rat at 10 μg/ml (0.5 μl/hr). The human HB-EGF infusion led to Evans blue extravasation (Fig. 2a) similar to the previous report on VEGF infusions^21^. Although the magnitude of enlargement was mild (1.5 fold), intraventricular infusions of HB-EGF resulted in a significant increase in the ventricular size (2.55 ± 0.09; mean ± s.e. in mm^2^; p = 0.0001), as compared to that of the vehicle control (1.65 ± 0.06 mm^2^), as assessed by cross-sectional area of the lateral ventricles (Fig. 2b,c). This experiment has demonstrated that intraventricular infusion of human soluble HB-EGF results in an elevated vascular permeability in the periventricular region of the lateral ventricle and mild ventriculomegaly.

### Mice expressing lacZ/β-gal overexpress human HB-EGF in the brain

**Figure 3:**
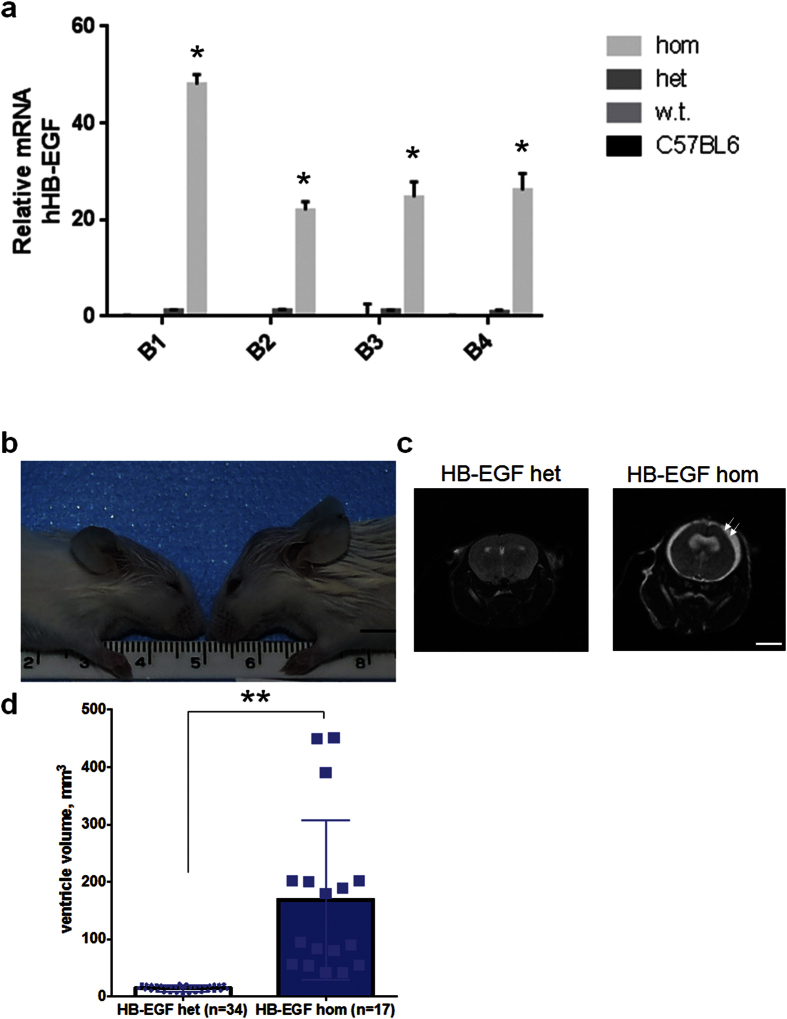
Characterization of mice expressing human HB-EGF (**a**) mRNA expression of human HB-EGF in four regions of the brain in age-matched C57BL6 and HB-EGF mutant animals. B1: olfactory bulb and prefrontal cortex, B2: motor cortex, B3: parietal and occipital cortex, and B4: cerebellum and visual cortex. Asterisk denotes statistical significance compared to the HB-EGF heterozygous animals at p < 0.05 by Tukey’s post-hoc test after ANOVA (P < 0.05). Note that no human HB-EGF transcript is detected in sibling wild type and C57BL6 mice. (**b**) A photograph showing a mutant phenotype with cranial doming in the HB-EGF homozygous mouse (right) as compared to the sibling wild type (left). n = 3 in each group for all the experiments in this figure (**a**,**b**). (**c**) Magnetic resonance images showing ventricular enlargement in the HB-EGF homozygous mutant. Het and hom denote HB-EGF heterozygous and homozygous genotype, respectively. Note that the wild type and HB-EGF heterozygous animals did not show a significant difference in head size or MRI scan of the brain ventricles. Note also that fluid accumulation in the subarachnoid space (arrows) and the fusion of lateral ventricles are evident in the HB-EGF homozygote at P60. (**d**) A bar graph with scattered data points exhibiting volumes of the ventricular system in mice carrying HB-EGF homozygous allele as compared to the heterozygous animals. n = 17 (littermate heterozygous) and n = 34 (homozygous) for all the experiments in this figure (**c**,**d**). The embryo derivation and MRI measurement of the brain ventricle for HB-EGF mice experiments were replicated twice by unrelated observers. Double asterisks indicate p < 0.01 by unpaired t test (two-tailed). Scale bars, 5 mm (**b**,**c**).

To further determine the localization of HB-EGF *in vivo*, we characterized mice expressing human HB-EGF (Fig. 3). The mRNA level of human HB-EGF in a brain region involving olfactory bulb and prefrontal cortex (B1) was significantly elevated (48-fold) in mice carrying human HB-EGF homozygous alleles as compared to the heterozygous control (p = 0.01). The mRNA expressions of human HB-EGF in brain regions involving motor cortex (B2), parietal and occipital cortex (B3), and visual cortex with cerebellum (B4) were significantly increased in mice carrying human HB-EGF homozygous alleles as compared to the heterozygous animals (p < 0.05). In age-matched C57BL6 and sibling wild type, human HB-EGF transcript was not detected (Fig. 3a). To identify the cranial doming of mice expressing human HB-EGF, the brain of HB-EGF mutants and wild type was visualized using magnetic resonance imaging. The brain scan suggests that mice carrying human HB-EGF with cranial doming have a significantly enlarged ventricle as compared to littermate heterozygous animals (Fig. 3c,d) with hemorrhage in the extra-axial space adjacent to the subarachnoid space (Supplementary Fig. S4). This experiment has exhibited that mice expressing lacZ/β gal display a significantly elevated level of human HB-EGF mRNA in the brain in the presence of ventriculomegaly and SAH.

### HB-EGF reporter is rarely detected in the SVZ

**Figure 4:**
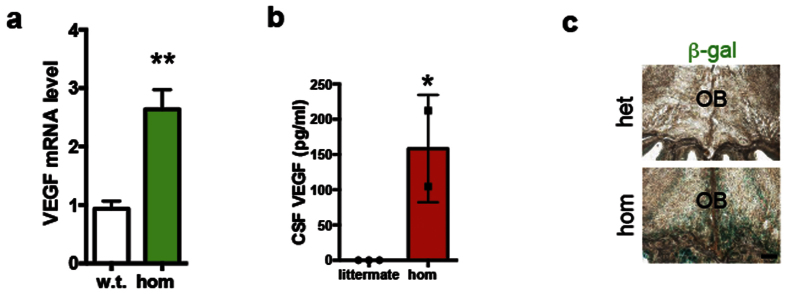
Expression of HB-EGF mRNA and the level of VEGF in the brain. (**a**) A bar graph showing the level of VEGF mRNA in the forebrain including olfactory bulb and rostral subependyma of mice over-expressing HB-EGF (homozygotes) as compared to wild type. n = 3 in each group in this figure. (**b**) A bar graph with scattered data points displaying the level of CSF VEGF (pg/ml) in littermate controls (n = 3) and in the HB-EGF homozygous mice (n = 2). (**c**) Localization of the β-gal reporting human HB-EGF protein in the OB (coronal sections) at P21. N = 3 in each group for all the experiments in this figure. Single and double asterisks in a and b denote p < 0.01 and p < 0.05 by unpaired t test (two-tailed), respectively.

To determine mRNA levels of human HB-EGF and VEGF in comparison with mouse endogenous HB-EGF focusing on target (exon 3) and off-target (exon 6) sequence, we assayed the brain tissue of mice carrying human HB-EGF homozygous alleles. The human HB-EGF mRNA was present in both heterozygous and homozygous brains (Supplementary Figs S2 and S3). Furthermore, the mRNA expression of VEGF in mice expressing human HB-EGF homozygous allele was significantly elevated by 2.6 ± 0.1 fold, compared to that of wild type controls as quantified by qRT-PCR (p = 0.001) (Fig. 4a and Supplementary Fig. S3). Consistent with this, the level of CSF VEGF protein (157.5 ± 48 pg/ml) was significantly increased (p = 0.02) in mice carrying HB-EGF homozygous allele as compared to the littermate control (0.1553 ± 0 pg/ml)(Fig. 4b).

To identify distributions of human HB-EGF reporter, β-gal, at baseline level, sagittal brain sections of the heterozygous mutant were assessed at early postnatal ages. At P1, the control brain demonstrated an increase of lacZ/β-gal in the region adjacent to the lateral ventricle with a detectable level of localization at nasal cavity. The stream of β-gal localization was detected in the region tangential to the lateral ventricle following the RMS (Supplementary Fig. S5a’-Sa”) and in the ependyma^22^ of the lateral ventricle (Supplementary Fig. S5a”) at P1. At P5 the localization of β-gal was clearly detected in the nasal epithelium but not in the OB of the heterozygous animals (Supplementary Fig. S5a”’). At P7, the localization of β-gal was evident in nasal cavity and Harderian gland (Supplementary Fig. S5a””) in mice carrying human HB-EGF heterozygous allele (Supplementary Fig. S5a). At P21, the localization of human HB-EGF reporter, β-gal, was observed in nasal epithelium and in the OB of the heterozygous animals. In mice carrying human HB-EGF homozygous alleles, however, an increased localization of β-gal was detected in the OB as compared to the heterozygous brain (Fig. 4c). This experiment has shown that mice over-expressing human HB-EGF also have the elevated VEGF mRNA, especially, in the forebrain region and a significant increase of VEGF protein in the CSF.

**Figure 5:**
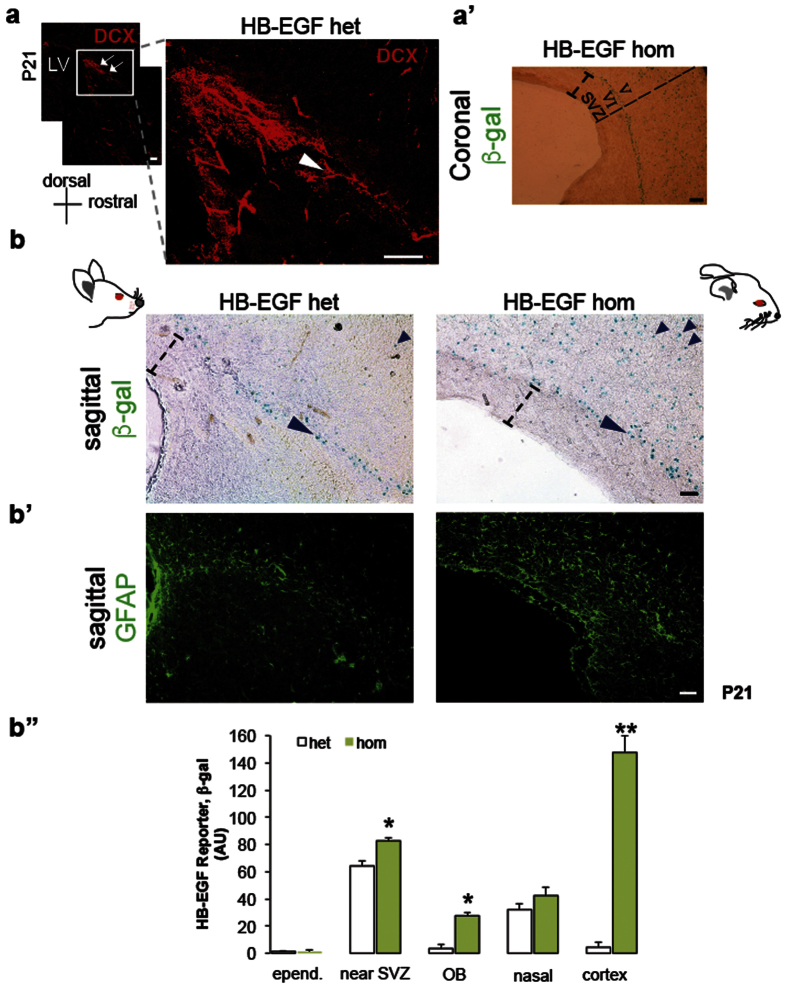
Localization of the HB-EGF reporter, β-gal, in the brain. (**a**) The RMS of mice with a HB-EGF heterozygous allele visualized with DCX immunofluorescence at P21. Arrows in the rectangular inset is magnified to the right, demonstrating the beginning trajectory of the RMS (arrow and arrowhead). (a’) Coronal section of mice carrying HB-EGF homozygous alleles at P21 displaying localization of β-gal from the cortical layer V–VI to the pia mater: within the SVZ and adjacent layer (I-bar), β-gal localization was rarely detected. Str denotes striatum. (**b**) Sagittal views displaying localization of the β-gal reporting human HB-EGF protein in the SVZ at P21. Note that the β-gal is specifically localized in the region where the RMS is observed (longer arrowhead) in the HB-EGF heterozygous brain (left), whereas a widespread distribution of the human HB-EGF (shorter arrowheads) is detected in the homozygous mutant brain (right). Longer arrowheads indicate a longitudinal orientation of transgene distribution tangential to the ventricular surface (left: longitudinal; right: longitudinal and radial). Note also that β-gal is distant from the ventricular surface more than 100 μm in mice expressing human HB-EGF (dashed I-bars). (b’) Adjacent sagittal sections stained with GFAP. Note that GFAP+ cells in the SVZ are specifically organized in an orientation longitudinally towards the OB of the HB-EGF heterozygous mutant (left). In the HB-EGF homozygous mutant, GFAP+ cells are widely distributed radially in the layer V–VI next to the SVZ (right). (b”) A bar graph displaying the regional distribution of HB-EGF reporter, β-gal, of the HB-EGF heterozygous and homozygous mutant mice at P21: epend denotes ependyma. N = 3 in each group for all the experiments in this figure (**a**,b”). Single (*) and double asterisks (**) represent p < 0.05 and p < 0.01, as compared to the heterozygous control, respectively, by unpaired t test (two-tailed). AU denotes arbitrary unit. Scale bars, 50 μm (**a**,b’).

To determine the β-gal localization in the OB of mice overexpressing human HB-EGF, the sagittal sections of the heterozygous mice were stained with anti-doublecortin (DCX) antibody. The RMS in the SVZ of the control mice showed tangential distribution of the DCX immunofluorescence with respect to the ventricular surface (Fig. 5a). Using this relatively rostral SVZ region, the distribution of human HB-EGF was assessed by lacZ/β-gal stain. The orientation of β-gal distribution starting from the SVZ showed three primary streams in saggital sections: one in longitudinal trajectory to the OB (RMS), the second in radial localization especially in the HB-EGF homozygous animals with hydrocephalus, and the third in ventrolateral direction (Supplementary Fig. S5b) similar to the lateral cortical stream described during development^6^. At P21, the β-gal localization was rarely detected in the ependyma and subependyma of the HB-EGF homozygote and heterozygote animals alike. In mice carrying human HB-EGF homozygous alleles, a widespread distribution of β-gal was observed in the cortical layer V–VI (Fig. 5a’), starting from the layer approximately 100 μm away radially from the lateral ventricle but rarely in the region next to the ventricular surface. The β-gal was distributed in both longitudinal and radial orientation in the cerebral cortex as compared to the control animals. The SVZ astrocytes in the RMS were also detected by GFAP+ immunofluorescence in mice overexpressing human HB-EGF as compared to the heterozygous controls in a sagittal view (Fig. 5b–b’). The spatial distribution as quantified by β-gal localization indicated that the HB-EGF localization was significantly elevated in the region near the SVZ following the RMS (p = 0.0026), in the olfactory bulb (p = 0.001), and in the cerebral cortex (p = 0.0001) of the homozygous animals as compared to the heterozygous control at P21. Unlike neonatal pups at P1 (Supplementary Fig. S5a”), β-gal localization was not detected in the ventricular surface (ependyma) of mice expressing human HB-EGF at P21 (Fig. 5b”). This experiment has determined that the human HB-EGF reporter, lacZ/β gal, is localized along the tangential migration from the SVZ to the OB in the control animals, while the HB-EGF reporter is widely distributed in the extra-SVZ throughout the cortical layers of the homozygous animal brain in the presence of ventriculomegaly.

**Figure 6:**
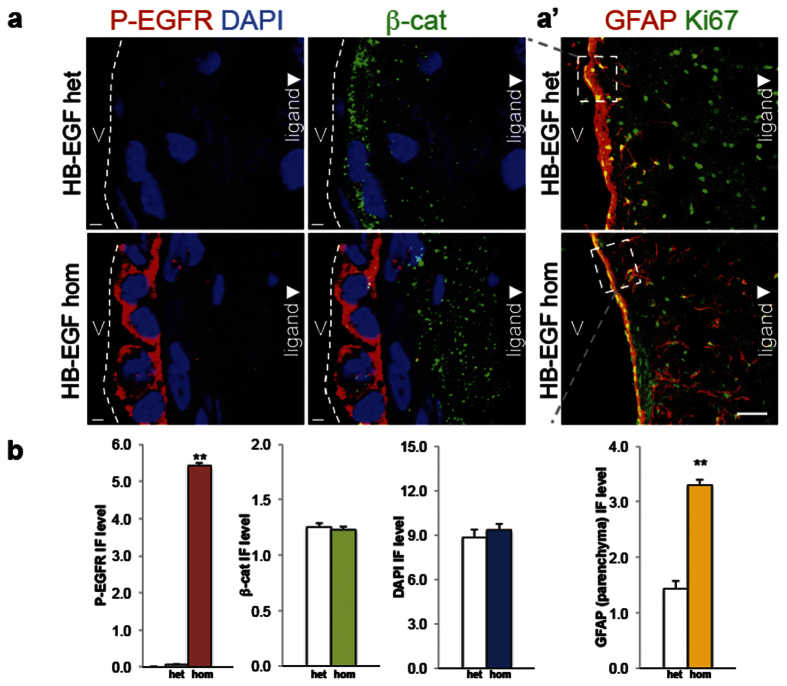
Response of cells on the ventricular surface to an EGFR ligand, HB-EGF. (**a**) Confocal micrographs displaying localization of phosphorylated EGFR (P-EGFR) immunofluorescence in the striatal side of the lateral ventricles (abbreviated, V) in mice carrying human HB-EGF heterozygous (upper) and homozygous (lower) alleles at P31. These sections are co-stained with β catenin (β-cat). Note that β-cat is diffusely detected in the SVZ but not on the ventricular surface of the HB-EGF homozygote as compared to the littermate controls. V denotes lateral ventricles. Arrowhead indicates an orientation in which EGFR ligand reporter, β-gal is located near the lateral ventricles. Dashed lines indicate apical membrane of the cells on the ventricular surface. (a’) Confocal micrographs exhibiting an adjacent coronal sections stained with GFAP and Ki67 in the SVZ of mice carrying human HB-EGF heterozygous (upper) and homozygous (lower) alleles at P21. V denotes lateral ventricles. Arrowhead indicates an orientation in which EGFR ligand reporter, β-gal is located near the lateral ventricles. Dashed rectangular insets correspond to the ventricular surface shown in a. (**b**) Bar graphs displaying the level of immunofluorescence (IF) in P-EGFR, β catenin, DAPI, and parenchymal GFAP shown in (**a–**a’). N = 3 in each group for all the experiments in this figure (**a**,**b**). Single (*) and double asterisks (**) represent p < 0.05 and p < 0.01, as compared to the heterozygous control, respectively, by unpaired t test (two-tailed). Scale bars, 10 μm (**a**) and 100 μm (a’), respectively.

To identify cells potentially responsive to HB-EGF in the SVZ of mice expressing human HB-EGF, we compared the striatal side of the lateral ventricle at P21. Unlike the previous report^17^, localization of the phosphorylated EGFR on the ventricular surface exposed to excess HB-EGF was observed in mice with hydrocephalus. In particular, the phosphorylated EGFR immunofluorescence was significantly elevated in the ventricular surface of mice overexpressing human HB-EGF as compared to the age-matched heterozygous control animals (Fig. 6a,b). Furthermore, GFAP+ SVZ astrocytes were located closely to the ventricular surface of the heterozygous animals while a significant increase of GFAP+ immunofluorescence in the parenchyma distant from the ventricular surface was detected in mice overexpressing human HB-EGF with homozygous alleles. A marker for cell proliferation, Ki67 was detected in the SVZ of both groups of animals but did not show a significant difference (Fig. 6a’–b). In this experiment we have identified that the phosphorylated EGFR is elevated in the ventricular surface of mice over-expressing human HB-EGF. Unlike the control SVZ, mice with hydrocephalus show that more GFAP+ SVZ astrocyte is detected in the parenchyma or in the extra-SVZ.

### VEGF inhibitor prevents VEGF-induced ventriculomegaly

**Figure 7:**
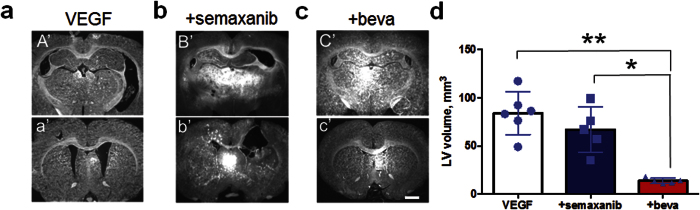
Effect of VEGF receptor (semaxanib) and ligand (bevacizumab) inhibition on ventricular size *in vivo*. (**a**) Coronal sections exhibiting a relatively caudal (A’) and rostral (a’) orientation of the rat brain infused with VEGF for 7 days and sacrificed on day 14. (**b**) Coronal sections displaying a relatively caudal (B’) and rostral (b’) orientation of the rat brain infused with VEGF for 7 days and treated with semaxanib for 7 days (VEGFR inhibitor). (**c**) Coronal sections showing a relatively caudal (C’) and rostral (c’) orientation of the rat brain co-infused with VEGF and bevacizumab for 14 days. (**d**) A bar graph with scattered plots demonstrating volume of the lateral ventricle (LV) for each infusion group. Single and double asterisks denote a statistical significance at p < 0.05 and p < 0.01, respectively, by Tukey’s post-hoc test after ANOVA. n = 5 (semaxanib and bevacizumab group); n = 6 (VEGF group). Scale bar, 1 μm (**a–c**).

In support of the previous reports^21^,^23^, the VEGF infusion at 25 μg/ml for 7 days results in ventriculomegaly and ependymal changes suggestive of altered junctional permeability (Supplementary Fig. S6). To determine whether blockade of VEGF receptor or ligand rescues experimental hydrocephalus independent of HB-EGF^21^,^23^, we examined the effect of intraventricular VEGF in combination with VEGFR2 blocker (semaxanib), or VEGF inhibitor (bevacizumab) on the size of lateral ventricles. The intraventricular infusion of VEGF for 7 days when treated with semaxanib for 7 days did not demonstrate a significant recovery of the ventricular enlargement (67 ± 11 mm^3^; p = 0.3) as compared to the VEGF infusion (84.5 ± 9 mm^3^). Unlike VEGFR2 inhibition, however, co-infusion of VEGF and bevacizumab for 14 days resulted in a significant decrease of the ventricular size (13.9 ± 1 mm^3^; p = 0.0001) as compared to the VEGF infusion alone (Fig. 7). Consistent with the previous report^24^, blockade of VEGF ligand and receptor (VEGFR2) through bevacizumab and semaxanib, respectively, prevented the VEGF-induced increase of polysialylated-neural cell adhesion molecule (PSA-NCAM)+ SVZ neuroblast in the RMS of rats treated with intraventricular infusions as compared to the rats infused with VEGF (Supplementary Fig. S7). In this experiment we demonstrated that VEGF inhibitor bevacizumab and VEGFR2 inhibitor semaxanib mitigate the mis-localization of PSA-NCAM+ neuroblast seen in the VEGF infused SVZ. However, semaxanib fails to rescue VEGF-induced ventriculomegaly in adult rats while co-infusion of VEGF and bevacizumab prevents it.

To further identify radial distribution of young neurons and radial glia in the postnatal brain with hydrocephalus independent of excess HB-EGF, we assessed the transmembrane protein 67 (TMEM67) mutant or Wistar polycystic kidney rats^25^ at an early postnatal age. At P17, the TMEM67 homozygous mutant rats demonstrated a reduced longitudinal distribution of the RMS as compared to sibling wild type (Supplementary Fig. S8a). When stained with a marker for radial glia and migrating neuroblast, the TMEM67 mutant with hydrocephalus displayed an increase of radial distribution of vimentin+ and DCX+ cells in the cerebral cortex as compared to the wild type animals (Supplementary Fig. S8b). In this experiment, we confirmed that the tangential migratory trajectory of the forebrain cells in the SVZ is altered and radial distribution of neuroblast and radial glia, rarely observed in the postnatal healthy brain, is detected in the Wistar polycystic kidney rats with hydrocephalus.

## Discussion

The results presented in this study support the idea that in order to regulate ventricular size HB-EGF confines its expression in the extra-SVZ of the cerebral cortex distant from the neurogenic niche in the postnatal forebrain. These findings enhance our understanding of neural cell distribution after birth and indicate that the HB-EGF-facilitated EGFR signaling exerts a highly controlled influence on the subpopulation of SVZ cells in a site-specific manner during postnatal period than previously thought. Excess HB-EGF led to ventriculomegaly in the presence of blood in the extra-axial space adjacent to the subarachnoid space. The brain scan through MRI and CSF collection exhibited that mice overexpressing human HB-EGF show PHH, suggestive of the SAH. This is an unusual observation that has not been reported in growth factor infusions such as in the VEGF-induced ventriculomegaly^23^. Genetic mutant and injection studies have reported hydrocephalus accompanying intracranial hemorrhage, subcortical heterotopia^26^, and IVH^4^ but rarely SAH. Using infusions, it has been claimed that thrombin-induced overexpression of TGF-β1 and activation of its downstream factors is a mechanism of hydrocephalus after SAH^20^.

**Figure 8:**
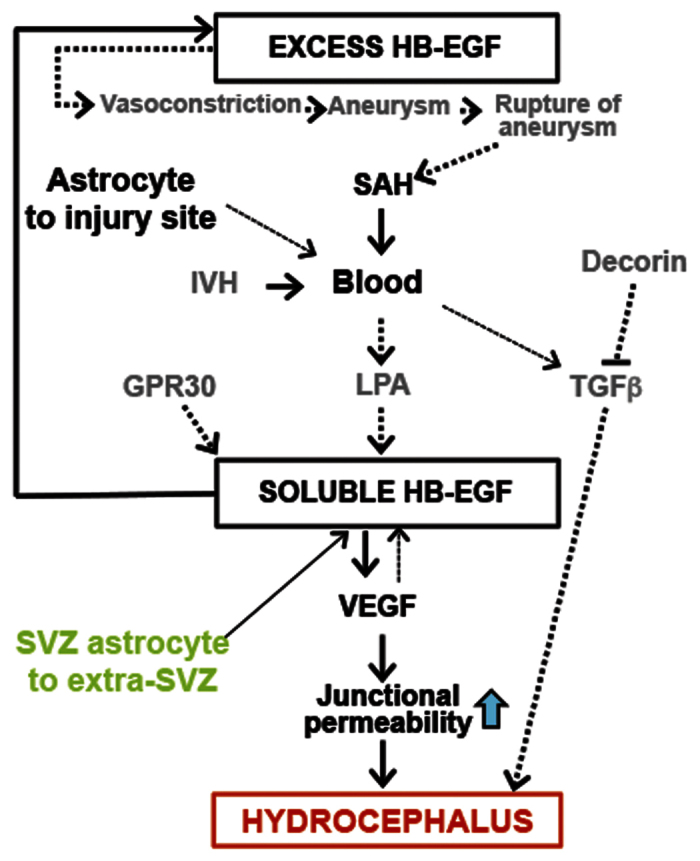
Summary of the hypothesized pathogenic mechanism. Rectangles represent the HB-EGF mediated hydrocephalus. Solid arrows indicate the causal relationship shown by the present study, while dashed arrows are hypothesized based on prior reports by others. Mice expressing human HB-EGF homozygous alleles were born with excessive HB-EGF, which can evoke aneurysmal SAH through vasoconstriction^9^,^10^. Blood from SAH releases LPA, which is shown to induce soluble HB-EGF via enzymatic cleavage of the membrane-bound precursor HB-EGF^48^. The soluble HB-EGF can activate EGFR expressing cells (upward arrow)^34^ or induces VEGF in epithelial cells such as endothelial and/or ependymal cell contacting fluid such as blood and/or CSF, respectively (downward arrow). The elevated level of VEGF can alter epithelial junctional protein complex via an increase of intracellular permeability, leading to leakage of fluid into the extraventricular space (parenchyma) and hydrocephalus^23^. An alternative route of TGFβ1 mediated hydrocephalus following SAH or IVH^3^,^20^,^29^ is shown in the right side, in which decorin has been shown to prevent it^31^. As an upstream causal factor, GPR30 has been shown to release HB-EGF^33^. As a consequence of bleeding, astrocytes can move to the injury site^49^ while, those residing in the SVZ (SVZ astrocyte B1 and B cell), show a displaced localization out of the SVZ in the brain with excess HB-EGF and hydrocephalus. IVH: intraventricular hemorrhage, CSF: cerebrospinal fluid, VEGF: vascular endothelial growth factor, LPA: lysophosphatidic acid, SAH: subarachnoid hemorrhage, TGFβ1: transforming growth factor beta 1, GPR30: G Protein-Coupled Receptor Homolog^50^,^51^,^52^,^53^.

In clinical reports, SAH alone is not sufficient to cause hydrocephalus. Why some cases of SAH lead to hydrocephalus but other cases remain in the absence of hydrocephalus is intriguing^27^. Our data, in addition to clinical risk factors such as IVH, admission level of consciousness, hypertension^28^, suggest that SAH when accompanying changes in migration and mislocalization of the PSA-NCAM+ neuronal progenitors is likely linked to acquired hydrocephalus. Consistent with TGFβ overexpression in the animals^29^, patients with SAH who developed communicating hydrocephalus displayed a significant elevation of CSF TGFβ levels^30^. This type of PHH has been shown to attenuate in animals when treated with TGFβ antagonist decorin^31^. Lysophosphatidic acid, a known causal factor of PHH^4^, is reported to mediate ectodomain shedding of membrane-bound HB-EGF to release soluble HB-EGF^32^. As an upstream causal factor, G Protein-Coupled Receptor Homolog, GPR30 has been shown to release HB-EGF^33^. In relation to other acquired hydrocephalus models such as induction by blood related factors, mice overexpressing HB-EGF reveal a hydrocephalic phenotype with SAH (Fig. 8).

The attempt to understand the regulation of cell migration by HB-EGF in the postnatal brain resulted in two unexpected data. Firstly, the HB-EGF reporter, β-gal was detected in the ventricular surface at birth (P1). At weaning age (P21), β-gal was rarely detected in the SVZ but highly visible in the cerebral cortex. The rare presence of the transgene reporter in the SVZ of mice expressing human HB-EGF suggests that it might be the subpopulation of cells expressing HB-EGF receptor but not HB-EGF that undergoes chemotactic migration. Unlike mice lacking EGFR as reviewed previously^12^, our data suggest that P-EGFR+ cells in the ventricular surface can be involved in migration by soluble HB-EGF circulating in the CSF^34^,^35^. This interpretation is bolstered by the observation that cells expressing EGFR migrate to the region of high EGFR ligand or HB-EGF through chemoattractive rather than chemorepellant mechanism^6^. The second one is that human HB-EGF reporter, β-gal, is detected in the vicinity of RMS and radial trajectory in the postnatal forebrain with hydrocephalus. Three independent studies reported that the RMS is either unaltered (GPCR) or impaired (hyh; tg737 orpk) in congenital models of hydrocephalus. The studies using these models of hydrocephalus did not report an alteration of radial migratory trajectory. Thus, the EGFR-mediated control of cell migration in the postnatal forebrain raises the question of the identity of the ligands mediating it. The cortical slice culture performed using HB-EGF in one side of the explant followed by EGFR immunohistochemistry provided compelling evidence that, to regulate migration in the developing forebrain, EGFR requires the ligand expression from the ventricular neurogenic niche to the marginal plate^6^.

An alteration of rostral migratory stream (RMS) is reported in animal models with hydrocephalus^36^. Unlike G-protein-coupled receptor-mediated hydrocephalus^37^, the mechanism(s) by which hydrocephalus elicits an alteration of migratory trajectory either in tangential or radial orientation or both by an identifiable underlying cause remain elusive^38^,^39^. Among several proposed mechanisms, it has been suggested that impairments of CSF flow and microtubule transport defect play a role in the development of abnormal migration^40^,^41^. This idea is supported by the observation that the orientation of neuroblast migration is correlated with the flow of CSF in postnatal rodent brains and that the transgenic 737 Oak Ridge Polycystic Kidney mutants show cell migration defects, especially in tangential orientation in the presence of hydrocephalus^41^.

Soluble HB-EGF, generated through ectodomain shedding, mediates cell migration via chemoattractive mechanism. The EGFR, however, is activated through phosphorylation either by soluble HB-EGF in the presence of heparin or heparin-like molecules^35^,^42^ or by transmembrane HB-EGF via juxtacrine activities^34^. It has been demonstrated that cell migration stimulated by an agonist of G protein-coupled receptor depends on EGFR phosphorylation by soluble HB-EGF. The heparin-like molecule such as heparan sulfate proteoglycan and matrix degrading enzyme (e.g. metalloproteinase) have been proposed to play a regulatory role in transmembrane HB-EGF shedding and cell migration^35^.

Given the prior observation of the developing telencephalon^6^ and the absence of β-gal in the OB of the heterozygous animals, however, EGFR-mediated migration does not primarily affect the RMS in healthy postnatal brains. At least two interpretations of these experiments can be conceived. The first idea is that three models of hydrocephalus^36^,^37^,^41^ are independent of EGFR and that either communicating or obstructive hydrocephalus regulates the RMS. Depending on communication of the CSF, therefore, the RMS is either unaltered following the flow of CSF or impaired due to obstruction against the CSF flow. A second interpretation suggests these two circumstances might be complementary and implies that EGFR ligands are necessary for EGFR to regulate radial migration but tangential migration is sustained by signaling molecule(s) other than HB-EGF in the postnatal brain with hydrocephalus.

This latter view lends further support if we look at another function regulated by HB-EGF: an ability to induce VEGF. The phosphorylation of VEGFR2 is increased in the SVZ neural progenitor cells of mice lacking VEGFR1 and in wild type mice infused with intracerebroventricular VEGF proposing that the proper RMS depends on endogenous VEGF protein in the postnatal brain^24^. The inconsistency between mice lacking VEGFR1 (RMS is slightly reduced but there was no hydrocephalus) and three other models of hydrocephalus (RMS is either reduced or unaltered) echoes the argument above for the regulation of neural migration^24^,^31^,^37^,^41^. Taken together they raise the prospect that HB-EGF may first act as a chemoattractant in the extra-SVZ of the postnatal forebrain to turn on the switch of chemotactic migration and facilitates the RMS by inducing endogenous VEGF in hydrocephalus.

Consistent with mice expressing human HB-EGF, the VEGF study in rats was to characterize the effect of VEGF-A_165_ (human isoform). As to the VEGF receptor, we have previously shown that VEGF-A_165_ infusion activates endogenous VEGF receptor by increasing the phosphorylated level of VEGFR2 immunofluorescence in the lateral ventricles^23^. VEGF is an angiogenic factor produced by choroid plexus of the cerebral ventricle and by the blood vessel. Both VEGF-A_165_/VEGF-A_121_ is elevated in hydrocephalus up to 1 ng/ml in the CSF^23^,^36^. VEGF-A_165_ is shown to affect the SVZ neurogenic activity and the number of dividing cells in the RMS^24^. It is a growth factor with two distinct functional identities for cell migration depending on concentration: an endogenous protein influencing the RMS of the normal mouse brain at 2.4 ng/d or 200 ng/ml in 200 μl at 0.5 μl/hr *in vivo*^24^; a permeability factor evoking monocyte extravasation through VEGFR1 inducing the monocyte chemotaxis at 1.3-5 ng/ml *in vitro*^43^. Although VEGF has several critical roles in endothelia, its potential function as a regulator of the RMS in the healthy brain and in hydrocephalus remains controversial. Our data and the previous reports by others (Supplementary Table S2) suggest that, unlike EGF^44^,^50^, HB-EGF induces VEGF and increases the redistribution of the SVZ cells radially in the cerebral cortex. In pathological conditions such as cancer, EGFR and VEGFR signaling are main targets to inhibit. In endothelial cells, it has been shown that VEGF also induces HB-EGF^45^. Our data supports the idea that VEGF and HB-EGF can be synergistic and form a positive feedback in the non-neoplastic brain with hydrocephalus.

Unlike the paucity of β-gal in the SVZ, GFAP+ astrocytes exhibited an increased distribution from the ventricular surface to the cortical layer following the RMS and radial orientation in the HB-EGF homozygous brain. The previous report also suggests that the EGFR-mediated chemoattractive migration is independent of cell type, in which chemotaxis can be elicited in astrocytes, neurons, and multipotent stem cells, once each cell type expresses sufficient numbers of EGFRs in the developing brain^6^. In adult mice, it has been shown that the subpopulation of GFAP+ SVZ astrocytes is neural stem cell^15^. Using EGF infusions, it has been shown that EGF responsive cells are EGFR+ but they are the mixed population of SVZ cells, primarily, transit amplifying cells and other types^44^. Our data support the interpretation that HB-EGF responsive cells with P-EGFR appear in the ventricular surface of the postnatal brain with hydrocephalus. Overall, we observed that HB-EGF induces VEGF. Using mice expressing HB-EGF, the localization of HB-EGF reporter is determined in the postnatal forebrain along the RMS without hydrocephalus. Expression of human HB-EGF results in a displaced localization of HB-EGF reporter, β-gal, in the region distant from the SVZ. Overexpression of human HB-EGF further elicits hemorrhage in the subarachnoid space, an increase of VEGF and phosphorylated EGFR in the presence of ventriculomegaly. VEGF inhibition in adult rats prevents ventriculomegaly and an increase of the SVZ neuroblast in tangential migratory stream. A radially distributed cell is increased in Wistar polycystic kidney rat, another model of hydrocephalus. Together, the present study supports the idea that excess HB-EGF promotes VEGF signaling and results in ventriculomegaly with SAH.

## Methods

### Cell culture

The source of cells is the brain of C57BL6 mice (age 50–75 days). All the mouse brain endothelial and non-endothelial cells used in the experiments were passages 4 to 12 (P4–P12). The purity of endothelial cell and the absence of mycoplasma contamination were authenticated^46^. The magnetic cell sorting scheme was applied. Briefly, the cells were labeled with a purified rat anti-mouse CD31/PECAM-1 antibody (BD Pharmingen™; San Jose, CA) with 1:50 ratio in the manual cell separation columns (MACS) buffer. Then, the cells were magnetically labeled with Goat Anti-Rat IgG MicroBeads (Miltenyi Biotec, Germany). Subsequently the cell suspension (500 μl) was transferred onto a column (MACS® separation columns). For CD31 (+) cells, either low glucose Dulbecco’s Modified Eagle’s Medium (DMEM: Gibco; Carlsbad, CA) containing 10% FBS, 10% Nu serum IV (BD Biosciences; San Jose, CA), 1 mg/1 ml porcine heparin, 1× antibiotic-antimycotic (ABAM), 6 ng/ml basic fibroblast growth factor (R&D Systems; Minneapolis, MN), and 1× Insulin-Transferrin-Selenium-A Supplement (ITS) was used. The negative control was a mixed population of fibroblast, non-neural and smooth muscle cell. For CD31 (−) cells, low glucose DMEM plus 10% Fetal Bovine Serum (FBS) and 1x ABAM was a growth medium. During culture period in the 5.0% CO_2_ incubator at 37 ± 0.4 °C, the dish was coated with fibronectin for CD31 (+) cells and the growth medium was changed every other day. Accutase (eBiosciences; San Diego, CA) and Trypsin-EDTA were used to detach CD31 (+) and CD31 (−) cells, respectively. Prior to reaching exponential growth phase or when 70% confluent as a primary culture for 14–21 days, 2 × 10^6^ cells from each 8 mm-diameter culture dish (BD Falcon; San Jose, CA) were transferred to 1.5 mm-diameter Nunclon™ 4-well plate (nunc™ Denmark; Rochester, NY) to promote cell-cell contact and proliferation. On average, 5 × 10^5^ cells were counted per each well. Both groups of CD31+ and CD31− cell were then treated with the serum free media overnight prior to the HB-EGF administration.

### Effect of exogenous HB-EGF

A stock of 50 μg/ml recombinant human heparin binding epidermal growth factor like growth factor (rhHB-EGF, R&D Systems; Minneapolis, MN) was reconstituted via sterile PBS containing 0.1% BSA to 10 μg/ml and 0, 10, and 100 ng/ml of each rhHB-EGF was added to 4-well plate. Either ribonucleic acid (RNA) samples or the cell culture supernatants were collected at 24 h and 72 h point. For a comparatively short-term response, both RNA sample and culture media were collected 1, 3, 6, and 12 h after the introduction of the same doses of rhHB-EGF. All medium samples were transferred to −20 °C and the extracted total RNA to −78 °C until the next step.

### Immunocytochemistry

The cells in the 1.5 mm-diameter 4-well plate and the 8 mm-diameter round culture dish were fixed in 4% paraformaldehyde for 20 min at −20 °C and rinsed three times for 5 min with PBS. Then, the blocking solution made of 0.3% goat serum with 0.1% 1× Triton-X. Then, the primary antibody diluted in PBS/5% BSA (1:50) was added for 1 h at room temperature. Next, the samples were rinsed well with PBS. The secondary antibody in PBS/5% BSA (1:100) was added for 1 h at room temperature and rinsed. Samples were then counterstained with 4′,6-diamidino-2-phenylindole (DAPI).

### Primers and qRT-PCR

A set of primers (SuperArray Bioscience Corp.; Frederick, MD) and sequence used are listed in Supplementary Table S1. The total RNA was extracted without Dnase-I treatment (Rneasy Mini Kit, Qiagen; Valencia, CA). The Sybr green super mix was used (iQ Sybr Green Mix, Bio-Rad; Hercules, CA) and quantitative reverse transcriptase polymerase chain reaction (qRT-PCR) conditions for thermal cycler were as follows: 3 min at 95 °C and 10 sec at 95 °C–45 sec at 55 °C (×40). For melting curve analysis, 1 min at 95 °C, 1 min at 55 °C, and 10 sec dwell time at 55 °C (×80) were employed.

### Mouse VEGF ELISA

Cell culture supernatants and CSF were collected and the VEGF protein level was assayed twice per well by enzyme linked immunosorbent assay (Quantikine mouse VEGF Immunoassay kit, R&D Systems; Minneapolis, MN). The arithmetic average over duplicate measurement was taken as a final VEGF concentration in the supernatants, where the lower and upper limit per the standard curve was set as 0 and 500 pg/ml, respectively. All ELISA 96-well microtiter plates were analyzed using Spectra Max 190 (Molecular Devices, Sunnyvale, CA).

### Surgical procedures

Animal surgery for infusions followed the previous report^23^. Briefly, male Sprague–Dawley (SD) rats (Charles River Laboratories) weighing about 250 g were used. Intraventricular infusion of recombinant human HB-EGF, VEGF-A_165_, and semaxanib was performed following published methods^21^,^23^. Anesthesia was induced and rats were positioned in a stereotaxic device (David Kopf, Tujunga, CA). Infusion cannulae of 5 mm length were used without modification (Durect; Brain Infusion Kit 2). Stereotaxic coordinates of 0.8 mm posterior and 1.4 mm lateral (left) to the bregma were used^20^. We infused with growth factors and drug using osmotic minipumps (Durect; 2002). The cannulae were cemented in place to minimize movement that could have damaged ventricular walls and adjacent structures. Briefly, three groups of infusions were designed with coiled PE50 tube: Group 1 (n = 6) of SD rats received intraventricular VEGF-A_165_ at 25 μg/ml for 7 days and sacrificed on day 14. Group 2 (n = 5) of SD rats received intraventricular VEGF-A_165_ at 25 μg/ml for 7 days (Day 1 to 7) and intraventricular semaxanib (100 ng/ml) for 7 days (Day 8 to 14). Group 3 (n = 5) of SD rats received intraventricular VEGF-A_165_ at 25 μg/ml with bevacizumab at 25 mg/ml for 14 days. Three groups of rats were sacrificed with intracardial perfusion through 4% paraformaldehyde (PFA) on day 14. To visualize vascular permeability in the rat brain infused with HB-EGF, 1% (weight/volume) Evans blue (Sigma Aldrich) dissolved in PBS was prepared, filtered, and injected in a volume of 4 ml/kg to the tail vein 1 h prior to sacrifice^47^. Brains were harvested, cryoprotected in sucrose, and sectioned at 10 μm thickness onto glass slides. Rodent use and procedures conformed to the National Institutes of Health (NIH) guidelines and were approved by the Institutional Animal Care and Use Committee at Boston Children’s Hospital. The male TMEM67 mutant animal (Wistar background strain; age P17) use and procedures also conformed to the NIH guidelines and were approved by the Institutional Animal Care and Use Committee at Indiana University Purdue University Indianapolis.

### Generation of mice expressing human HB-EGF

A pIRES1hyg plasmid by Clontech (6061-1, Mountain View, CA) was used as the starting agent to create a vector for the HB-EGF overexpression transgene (Supplementary Fig. S1). The pIRES1hyg contains the internal ribosome entry site (IRES) of the encephalomyocarditis virus (ECMV) allowing for two open reading frames from one mRNA. The plasmid also has a hygromycin B resistance for selection. The expression cassette contains human cytomegalovirus (CMV) major immediate early promoter/enhancer followed by a synthetic intron, multiple cloning site (MCS) known to enhance the stability of the mRNA. The ECMV IRES is followed by the hygromycin B phosphotransferase gene, and the poly adenylation signal for the bovine growth hormone. This allows ribosomes to enter bicistronic mRNA either at the 5′ end to translate the gene of interest or at the ECMV IRES to translate the antibiotic resistance marker. The exon 3 of HB-EGF genome is replaced with soluble human HB-EGF by digesting the pIREShygHB-EGF AscI#12 with BamHI to release the insert and ligate with the PCR product HB-EGF Bam and HB-EGF 1–150 rev to generate pIREShygHB-EGF 1–150 AscI. This product was then digested with NotI and XbaI to open the plasmid again to be able to insert NTR and LacZ into the plasmid. A 4.7 kb fragment was inserted by ligating with SmaI to create a pIREShygHB-EGF1-150AscI NTR-LacZ. The transgene was transformed to pIREShygHB-EGF XhoI->AscI NTR-LacZ. Then the transgene was sent to the animal facility for blastocyst injection by cutting the final product with NruI and AscI. After receiving the animals with the transgene, confirmation was made by PCR product of the LacZ marker. For lacZ/β-gal assay in histology, X-gal detection of β-Galactosidase Kit (ClonTech) was used. The HB-EGF transgene was incorporated in the CD-1 background strain and male mice with postnatal day 1, 5, 7, 21, and 50–75 were used.

### Magnetic resonance imaging (MRI)

The animals were anesthetized with 1% isoflurane in an anesthetic chamber and once unconscious a snout mask was secured over the mouth and nose of the animal. The animal was placed supine in the MRI coil apparatus. Once secured in the MRI coil, the animal was gently placed in an 8 Tesla magnetic field using the MRI machine at small animal imaging facility of Harvard Medical School. T2-weighted images were obtained using a Bruker 8.5 T DRX vertical bore micro-imaging system (Bruker Instruments, Billerica, MA) with TR = 1000 ms, TE = 8.8 ms with a slice thickness of 0.75 mm, matrix size of 128 × 128 and a 2.56 × 2.56 cm^2^ field of view. The volumes of ventricles were determined using software developed in Mitch Albert’s lab of Brigham and Women’s Hospital where the CSF compartments were segmented on each MRI slice and extrapolation of the imaging data is calculated producing a picture representation and volumetric measurement of each animal.

### Immunofluorescence

Slides were incubated with primary antibodies diluted in blocking solution overnight at 4 °C, rinsed, and incubated with the secondary antibodies for 1 h at room temperature. Primary antibodies were the following: rabbit anti-CD31 (1:100, BD Biosciences), rabbit anti- VEGFR2 (1:200, Abcam), rabbit anti-DCX (1:200, Cell Signaling), mouse anti-GFAP (1:200, Abcam), mouse anti-PSA-NCAM (1:200, Millipore), mouse anti-α smooth muscle actin (1:500, Sigma), mouse anti-acetylated α tubulin (1:1000, Sigma), rabbit anti-β catenin (1:500 Abcam), rabbit anti-phosphorylated EGFR (1:200, Cell Signaling), and rabbit anti-Ki67 (1:200, Abcam). For nuclear staining 4′,6-diamidino-2-phenylindole (DAPI) 500 ng/ml (Sigma) was used. Secondary antibodies were Alexa Fluor dye-conjugated goat anti-mouse or goat anti-rabbit IgG or donkey anti-goat IgG (diluted 1:200 in blocking solution, Jackson ImmunoResearch or Invitrogen). Confocal images were taken on LSM 510 META NLO (Carl Zeiss MicroImaging, Inc., Thornwood, NY, USA). The following filter setting is applied: FITC Ch2 band pass (BP) 500–550 IR Texas Red Ch3: long pass (LP) 595 DAPI Ch2: BP 435–485 IR. For low magnification fluorescence micrographs Nikon E800 and 80i upright microscopes with Spot RT cooled CCD cameras were used.

### Image Analysis

Images of histological sections were analyzed at NIH ImageJ to compare ventricular size. For intraventricular infusion dataset, rat brains with incorrect cannula placement were excluded. The DAPI stained sections was processed through edge detection, binarization, and pixel count. This enabled to calculate pictorial element or cross-sectional area. Serial sections with 100 μm interval involving forebrain, midbrain, and hindbrain from three animals per genotype were used. Random number was generated to blindly allocate each slide for quantification of ventricular size and immunofluorescence.

### Statistical analysis

Power analysis was conducted using Statgraphics Centurion software (ver. 6, Warrenton, VA) to determine the sample size of the brain scan dataset based on the preliminary measure of ventricle volume by MRI. Statistical analyses were performed using Prism (version 5, GraphPad Software Inc.). Normality of data distribution was tested using the F-test for unequal variance. Normally distributed data were analyzed using Student’s t-test and ANOVA when comparing two and more than two groups, respectively. Tukey’s multiple comparisons as a posthoc test was used with multiplicity adjusted P value for each comparison. Non-normally distributed data were analyzed using the nonparametric Mann-Whitney test and Kruskal-Wallis with Dunnett’s post hoc tests when comparing two and three data groups, respectively. Data were expressed as average ± standard error (s.e.) of the mean (SEM) and were considered significant at the p ≤ 0.05 level.

## Additional Information

**How to cite this article**: Shim, J. W. *et al*. Excess HB-EGF, which promotes VEGF signaling, leads to hydrocephalus. *Sci. Rep*. **6**, 26794; doi: 10.1038/srep26794 (2016).

## Supplementary Material

Supplementary Information
