# Supplementary material for: Excess HB-EGF, which promotes VEGF signaling, leads to hydrocephalus
Source: Sci Rep. 2016 May 31;6:26794. doi: 10.1038/srep26794 (PMC4886677; doi:10.1038/srep26794)
Supplement: Supplementary Information [file srep26794-s1.pdf]

Supplementary Information

Title: Excess HB-EGF, which promotes VEGF signaling, leads to hydrocephalus

Joon W. Shim,[a,c,1*] Johanna Sandlund,[b,2,3] Mustafa Q. Hameed,[a,f] Bonnie Blazer-Yost,[c] Feng C. Zhou,[d] Michael Klagsbrun,[b,e] Joseph R. Madsen[a]

[a]Department of Neurosurgery, Boston Children's Hospital and Harvard Medical School, Boston, MA 02115, USA
[b]Vascular Biology Program, Boston Children's Hospital and Harvard Medical School, Boston, MA 02115, USA
[c]Department of Biology, Indiana University Purdue University, Indianapolis, IN 46202, USA
[d]Department of Anatomy and Cell Biology, Indiana University School of Medicine, Indianapolis, IN 46202, USA
[e]Department of Surgery and Pathology, Boston Children's Hospital and Harvard Medical School, Boston, MA 02115, USA
[f]Department of Neurology, Boston Children's Hospital and Harvard Medical School, Boston, MA 02115, USA

Present address

[1]Department of Medicine, Boston University School of Medicine, Boston, MA 02118, USA
[2]Department of Pathology, Stanford University School of Medicine, 300 Pasteur Drive L235, Stanford, CA 94305, USA
[3]Clinical Microbiology Laboratory, Stanford University Medical Center, 3375 Hillview Avenue Palo, Alto, CA 94304, USA

Supplementary Table S1 list of primers

Gene name	GenBank #	Reference Position	Band size	Sequence
Human HB-EGF	NM_001945	516	121	*
Mouse HB-EGF (exon3)	NM_010415	513-690	324	ctttctcctccaagccacaa tgagaagtcccacgatgaca
Mouse HB-EGF (exon1-4)	NM_010415	46-603	527	accttcaagggctggagtg ttcttccctaacccctttcc
Mouse HB-EGF (exon6)	NM_010415	2027	89	*
Mouse VEGF	NM_009505.3	1839-1857	191	*
Mouse GAPDH	NM_008084.2	962-983	128	*

* Refer to Qiagen/SuperArray (Bethesda, MD)

Supplementary Table S2 Summary of growth factor infusions

Craig et al (1996)	Doetsch et al (2002)	Kuhn et al (1997)	Kuhn et al (1997)	Johanson et al (1999)	Harrigan et al (2002)	Warner-Schmidt & Duman (2007)	Current study
**EGF**	**EGF**	**EGF**	**FGF-2**	**FGF-2**	**$VEGF_{165}$**	**$VEGF_{164}$**	**HB-EGF; $VEGF_{165}$**
Bind to heparin?<br>No	No	No	Yes	Yes	Yes	Yes	Yes
[μg/ml]<br>33		[μg/ml]<br>30	[μg/ml]<br>30	[μg/ml]<br>1<br>0.5<br>0.25	[μg/ml]<br>25<br>5<br>1	[μg/ml]<br>10	[μg/ml]<br>10;<br>25
mouse	rat	rat	rat	rat	rat	rat	rat
Flow rate<br>0.5 μl/hr	0.5 μl/hr	0.5 μl/hr	0.5 μl/hr	0.5 μl/hr	1.0 μl/hr	1.0 μl/hr	0.5 μl/hr
[ng/d]<br>400	[ng/d]<br>400	[ng/d]<br>360	[ng/d]<br>360	[ng/d]<br>12<br>6<br>3	[ng/d]<br>600<br>120<br>24	[ng/d]<br>240	[ng/d]<br>120;<br>300
How long?<br>6 d	6 d ON/1 d OFF<br>6 d ON/2 d OFF	14 d	14 d	2, 3, 5 d<br>10-12 d	7 d	7 d ON/7 d OFF<br>7 d<br>14 d	14 d;<br>7 d ON/7 d OFF

d denotes day; ON means pump is infusing infusate; OFF means pump is not infusing.

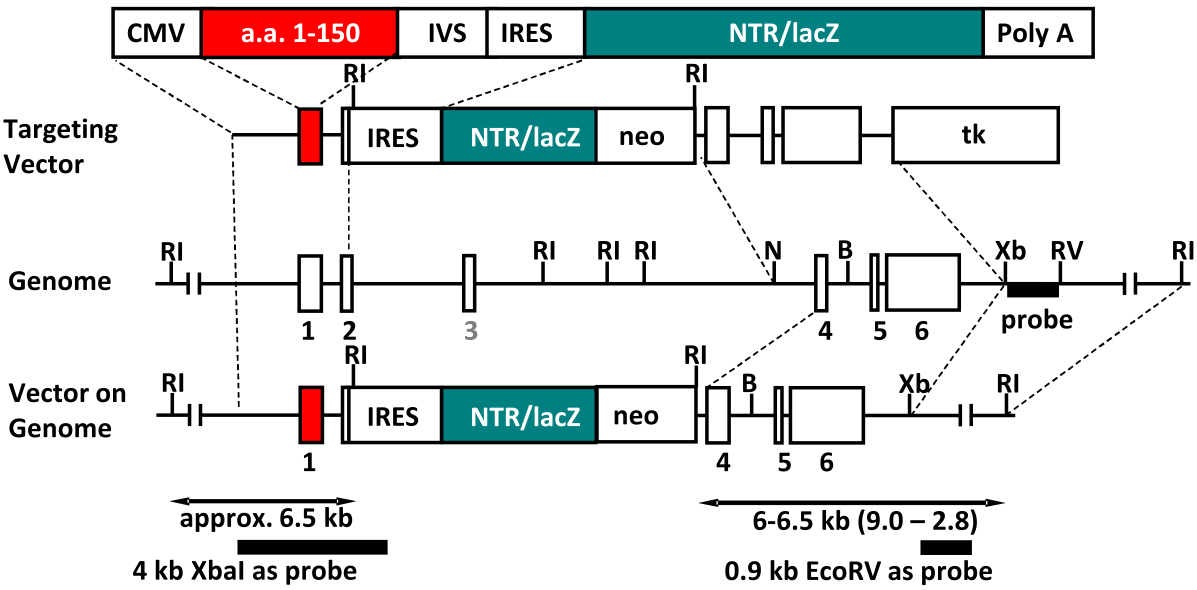

Supplementary Figure S1 A diagram exhibiting applied plasmid and transgene designed to express human HB-EGF (exon 3)

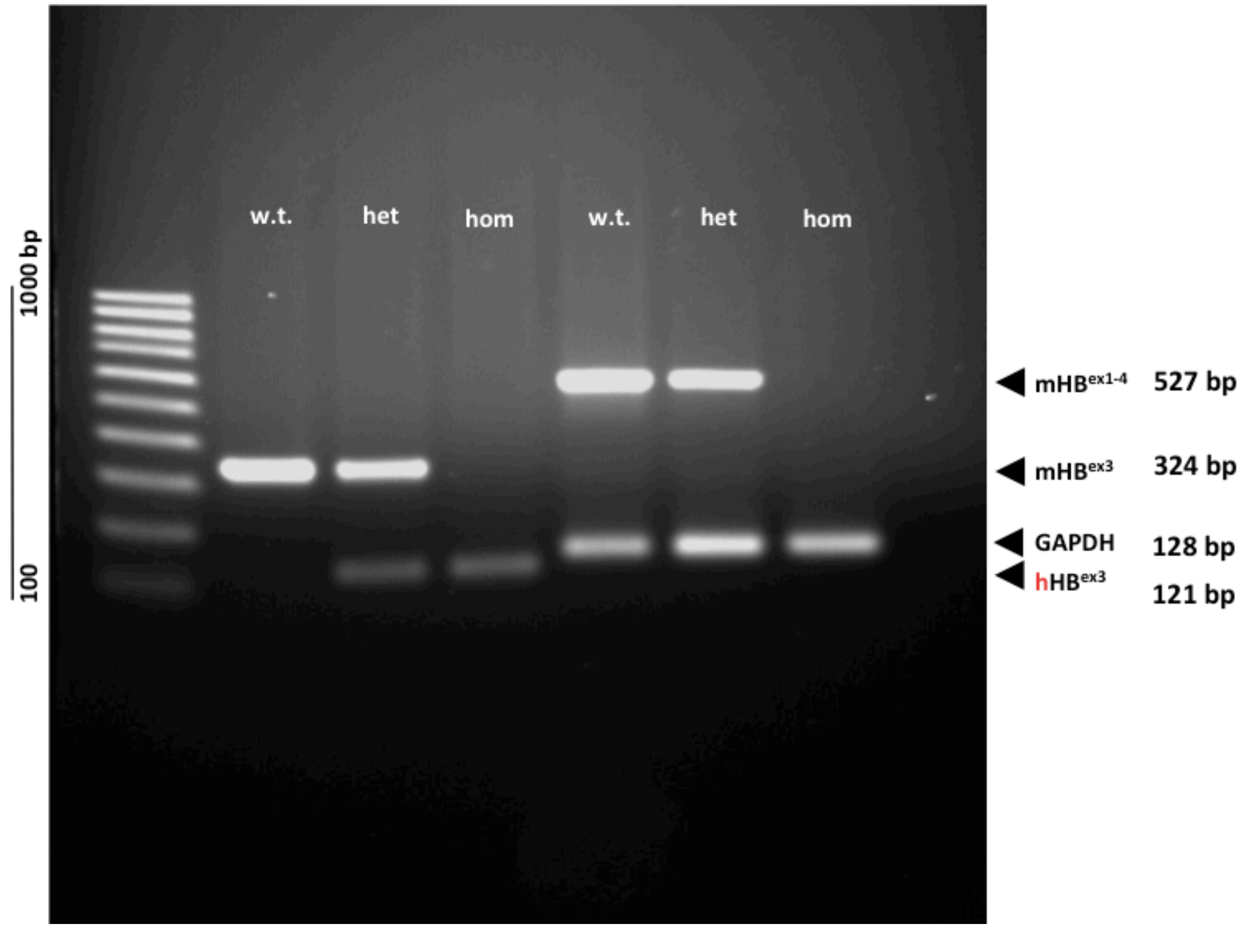

Supplementary Figure S2 Agarose gel showing RT-PCR result of each genotype with three primers sensing exon 1-4 (527 bp), exon 3 transcript of mouse HB-EGF (324 bp), and exon 3 transcript of human HB-EGF (121 bp) with GAPDH as housekeeping gene (128 bp). W.t., het, and hom denote wildtype, heterozygous, and homozygous mutant, respectively. mHB-EGF and hHB-EGF denote mouse and human HB-EGF, respectively. bp denotes base-pair.

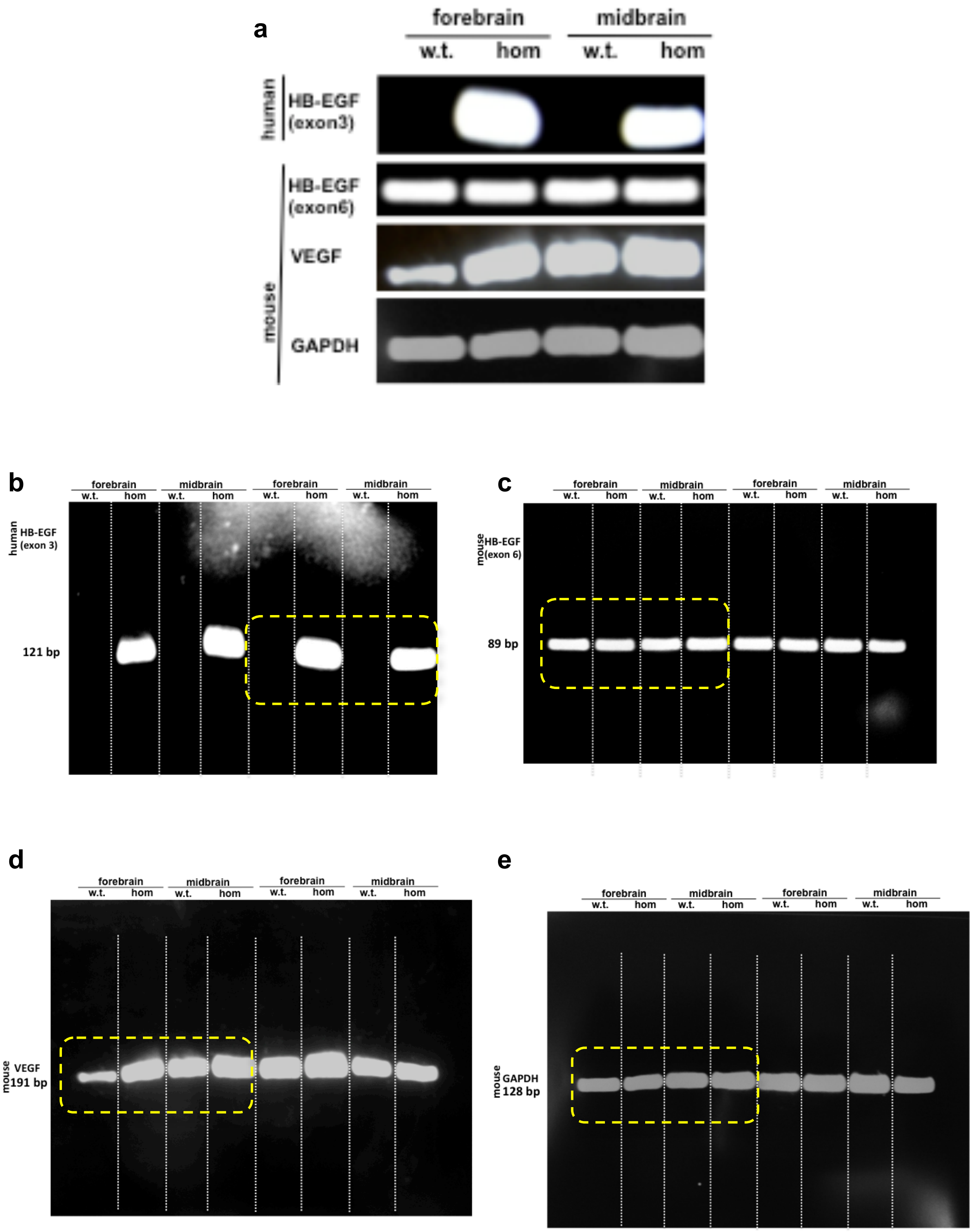

Supplementary Figure S3 Agarose gels displaying RT-PCR of the forebrain and midbrain region in the wild type animals and mice expressing human HB-EGF homozygous allele. (a) mRNA expression of human and mouse HB-EGF, VEGF, and GAPDH in the homozygote and wild type control animals (cropped from original images shown in c-f (b) Full-length gel showing human HB-EGF mRNA expression in the homozygote (c) Full-length gel displaying mouse HB-EGF expression in an off-target region (exon 6) (d) Full-length gel exhibiting elevated mouse VEGF mRNA level in the homozygote as compared to the wild type forebrain (e) GAPDH as internal reference. n=2/genotype (pair #1: lane 1-4, pair #2: lane 5-8). Dotted rectangles represent the cropped bands shown in a. Raw images visualized with Ethidium bromide in b-e

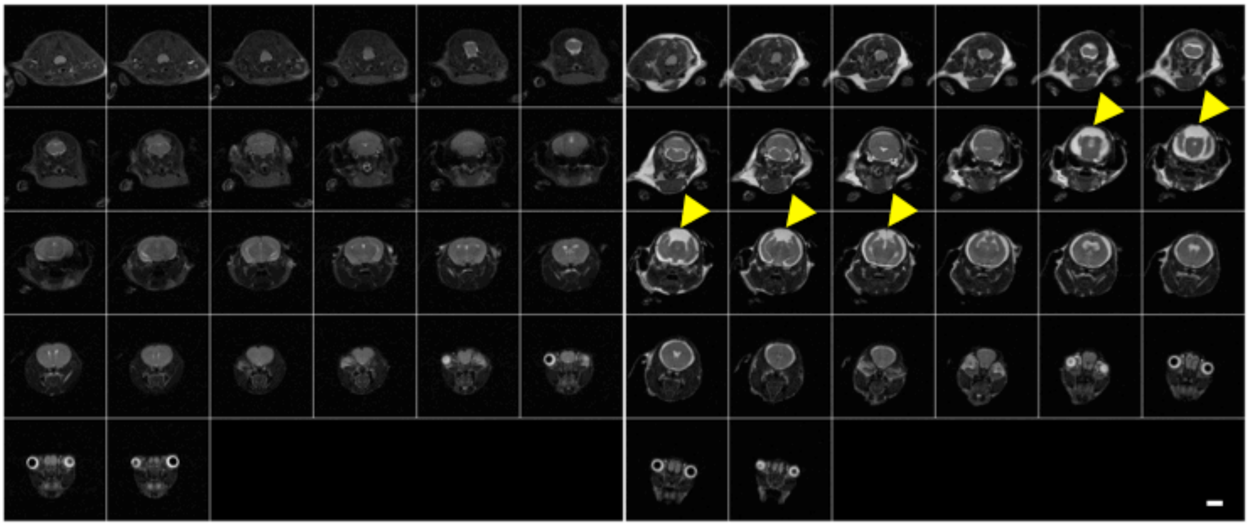

Supplementary Figure S4 The representative magnetic resonance image (MRI) of the mouse carrying human HB-EGF heterozygous (left) and homozygous (right) allele in coronal orientation at postnatal day 60 (P60): relatively caudal to rostral scan from the top left to the bottom. Note that the HB-EGF homozygote displayed the accumulation of cerebrospinal fluid in the subarachnoid space (arrowheads) and the cerebral ventricular system. Scale bar, 5 mm.

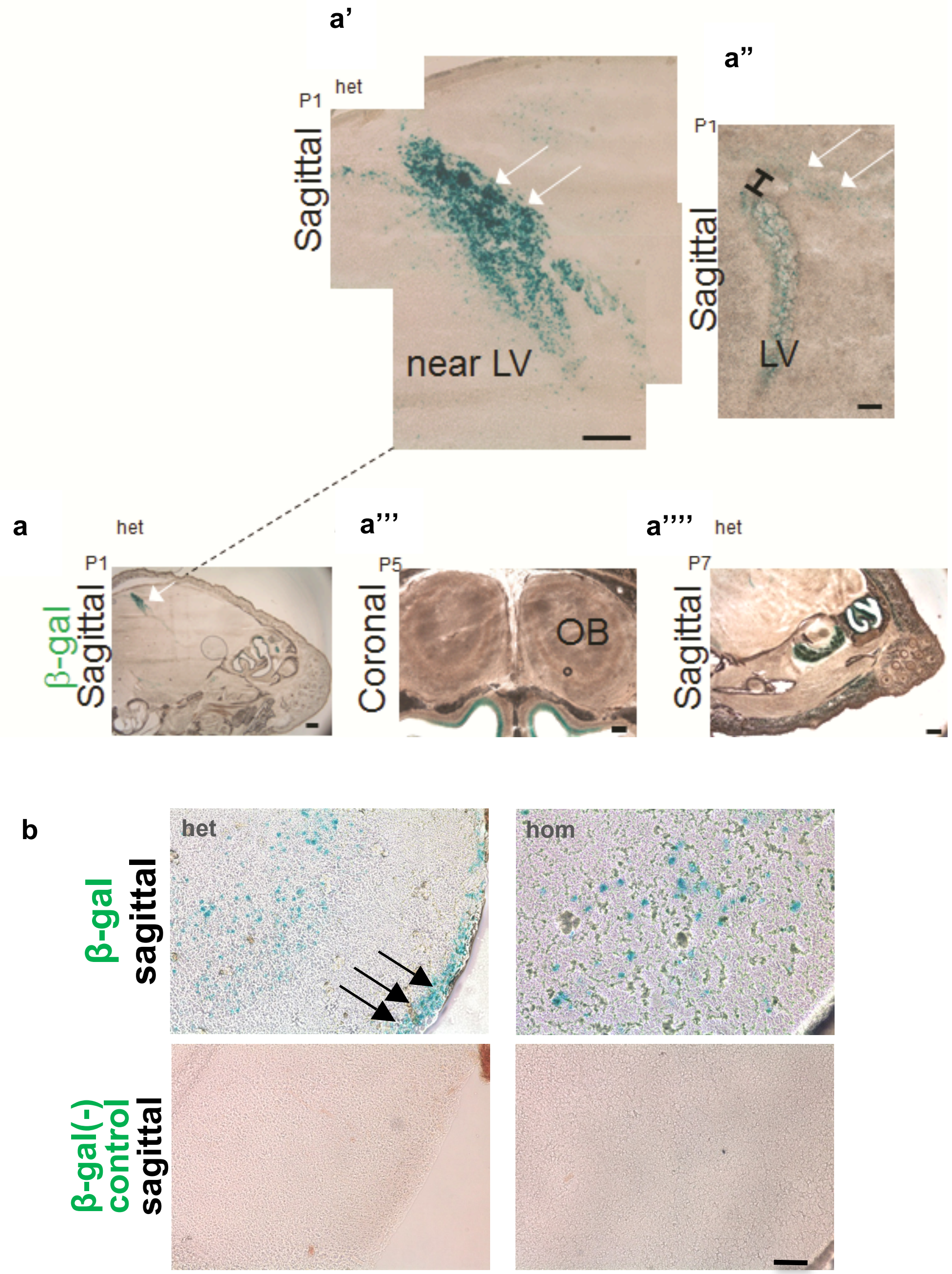

Supplementary Figure S5 (a) Localization of the transgene reporter, β-gal (green), in the HB-EGF heterozygous brain at P1, P5 (a'''), and P7 (a''''), respectively. Arrow indicates β-gal stream found in the vicinity of the lateral ventricle. This is magnified in B'. Adjacent sagittal sections displaying β-gal localization in the lateral ventricle (LV) and in a region dorsal and tangential to the LV. I-bar represents the distance between the LV and the tangential stream of the β-gal localization (a"). OB denotes olfactory bulb. Double arrows indicate a stream of tangential β-gal following the RMS (a'-a"). (b) Distribution of the β-gal in the HB-EGF heterozygous (left) and homozygous (right) brain at P21 in the ventrolateral direction reported previously [20]. Note that a dense ventral β–gal stream (arrow) is lost in the homozygote. Scale bars, 1 mm (a & a''''); 50 μm (a', a", a''', and b).

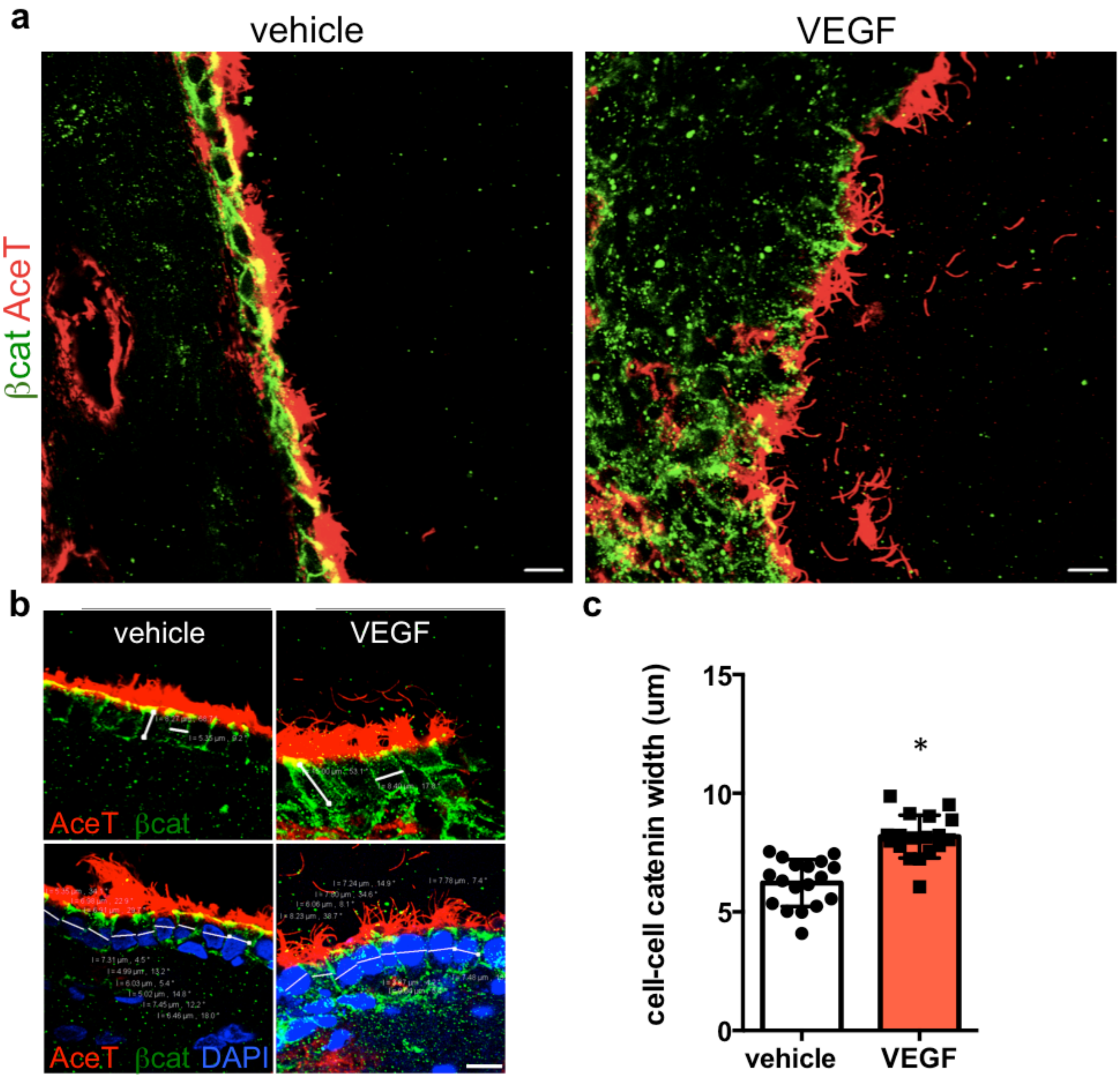

Supplementary Figure S6 Effect of intraventricular VEGF infusions on ependyma: (a) Micrographs demonstrating lateral walls of the lateral ventricle in the rostral SVZ of adult rats infused with vehicle (left) and VEGF at 25 µg/ml with the rate of 0.5 µl/hr for 7 days stained with β catenin (βcat) and acetylated α tubulin (AceT). (b) Micrographs displaying the size of basolateral βcat span indicative of altered intracellular junction on the ventricular surface: an apparent increase of width between one catenin immunofluorescence to another in an orientation along the ventricular surface is evident in the VEGF infused ependyma (c) A bar graph with scattered data exhibiting intracellular β catenin width in the vehicle (6.22±0.2 µm) and VEGF infused brain (8.17±0.2 µm). Asterisk denotes a statistical significance by Mann-Whitney U test at $p<0.05$ (n=18 cells on the ventricular surface from two animals per each infusion group). Scale bars, 10 µm (a-b).

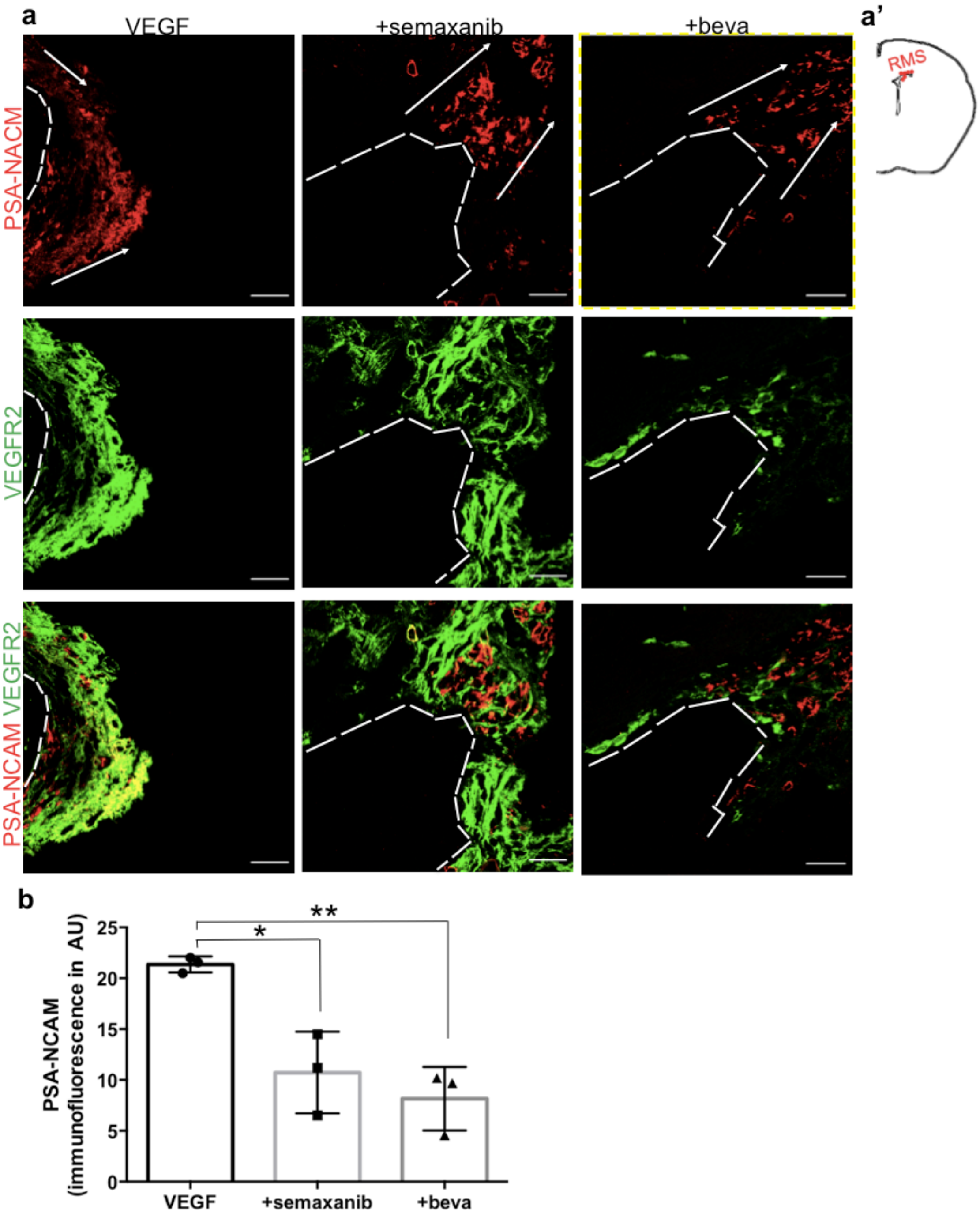

Supplementary Figure S7 Effect of VEGF receptor or ligand inhibition on SVZ neuroblast in the RMS: (a) Immunofluorescence micrographs displaying PSA-NCAM+ neuroblast adjacent to VEGFR2+ cells residing in the ependyma and subependyma of the rats treated with VEGF (left), VEGF with semaxanib (middle), and VEGF and bevacizumab (right). Arrows indicate an orientation of PSA-NCAM+ chains of SVZ neuroblast. Dashed line indicates ventricular surface. (a') Cartoon of the corresponding region visualized in A. (b) A bar graph showing the PSA-NCAM+ immunofluorescence in the region shown in A. Single and double asterisk denote a statistical significance at $p<0.05$ and $p<0.01$, respectively, by Tukey's post-hoc test after ANOVA ($P < 0.05$); scale bar, 20 μm (a)

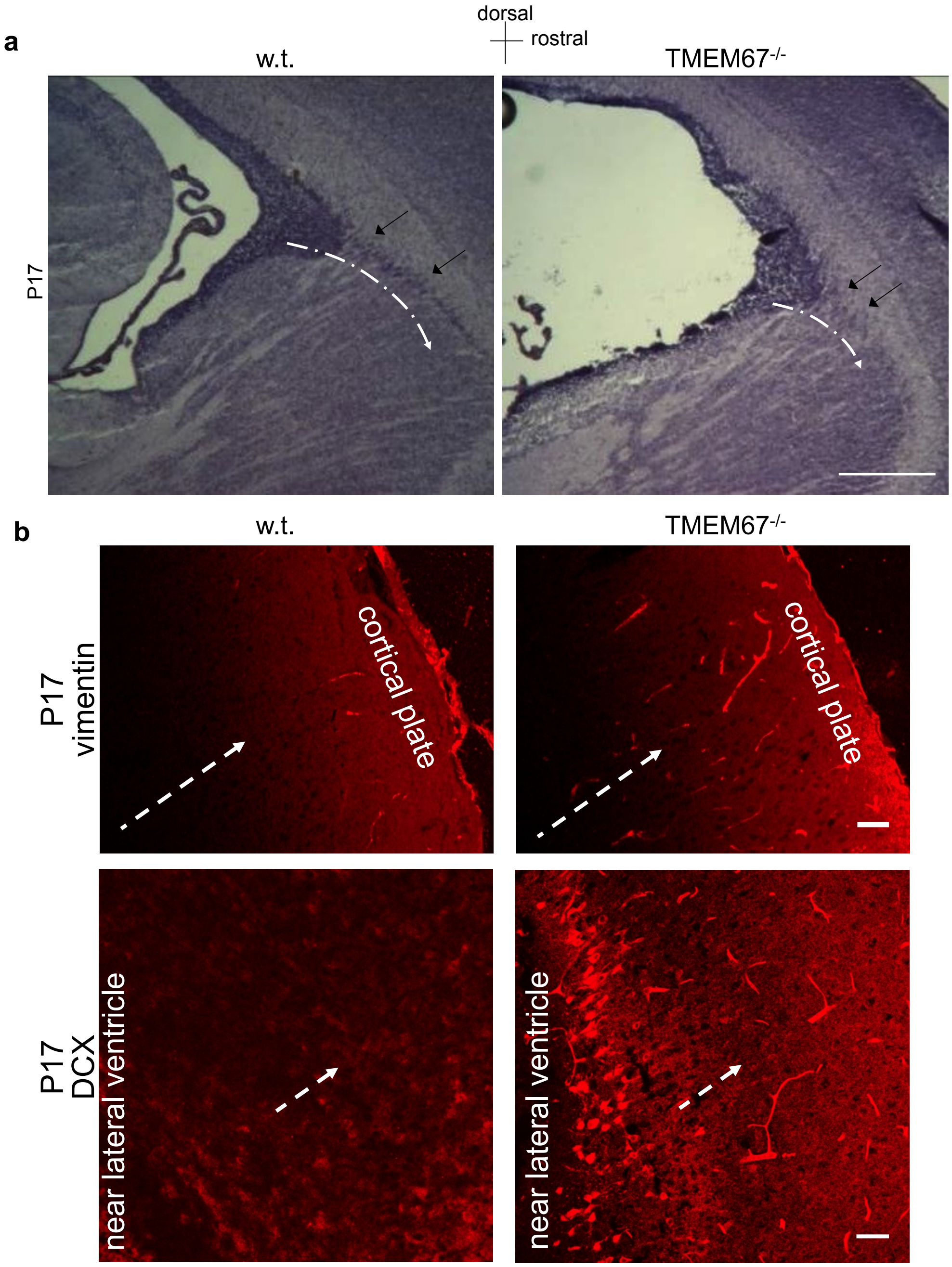

Supplementary Figure S8 Distribution of radial glia and young neurons in the SVZ and cerebral cortex of TMEM67 mutant rats with hydrocephalus: (a) Sagittal sections displaying cells reported to migrate tangentially from subventricular zone (SVZ) of the lateral ventricle to the OB. A curved arrow indicate the trajectory of RMS. Solid arrows indicate a reduced H&E stain the RMS of the TMEM67-/- mutant brain as compared to the wild type. (b) Confocal micrographs exhibiting an enhanced radial migration of vimentin+ radial glia (top) and DCX+ neuroblast in an radial orientation of the TMEM67-/- mutant brain as compared to the wild type. Dashed arrows indicate the orientation from the lateral ventricular surface towards the cortical plate. Scale bars, 500 µm (a) and 50 µm (b)
